# High Concentration of Vitamin E Decreases Thermosensation and Thermotaxis Learning and the Underlying Mechanisms in the Nematode *Caenorhabditis elegans*

**DOI:** 10.1371/journal.pone.0071180

**Published:** 2013-08-12

**Authors:** Yiping Li, Yinxia Li, Qiuli Wu, Huayue Ye, Lingmei Sun, Boping Ye, Dayong Wang

**Affiliations:** 1 Key Laboratory of Developmental Genes and Human Diseases in Ministry of Education, Medical School of Southeast University, Nanjing, China; 2 College of Life Sciences and Technology, China Pharmaceutical University, Nanjing, China; Imperial College London, United Kingdom

## Abstract

α-tocopherol is a powerful liposoluble antioxidant and the most abundant isoform of vitamin E in the body. Under normal physiological conditions, adverse effects of relatively high concentration of vitamin E on organisms and the underlying mechanisms are still largely unclear. In the present study, we used the nematode *Caenorhabditis elegans* as an *in vivo* assay system to investigate the possible adverse effects of high concentration of vitamin E on thermosensation and thermotaxis learning and the underlying mechanisms. Our data show that treatment with 100–200 µg/mL of vitamin E did not noticeably influence both thermosensation and thermotaxis learning; however, treatment with 400 µg/mL of vitamin E altered both thermosensation and thermotaxis learning. The observed decrease in thermotaxis learning in 400 µg/mL of vitamin E treated nematodes might be partially due to the moderate but significant deficits in thermosensation, but not due to deficits in locomotion behavior or perception to food and starvation. Treatment with 400 µg/mL of vitamin E did not noticeably influence the morphology of GABAergic neurons, but significantly decreased fluorescent intensities of the cell bodies in AFD sensory neurons and AIY interneurons, required for thermosensation and thermotaxis learning control. Treatment with 400 µg/mL of vitamin E affected presynaptic function of neurons, but had no remarkable effects on postsynaptic function. Moreover, promotion of synaptic transmission by activating PKC-1 effectively retrieved deficits in both thermosensation and thermotaxis learning induced by 400 µg/mL of vitamin E. Therefore, relatively high concentrations of vitamin E administration may cause adverse effects on thermosensation and thermotaxis learning by inducing damage on the development of specific neurons and presynaptic function under normal physiological conditions in *C. elegans*.

## Introduction

Vitamin E, a generic term for tocopherols and tocotrienols containing a group of eight lipid soluble substances with a chromanol ring and a saturated or unsaturated carbon side chain, has been widely studied for decades [1]. Natural vitamin E has potent neuroprotective function against the neurotoxicity induced by toxicants such as manganese, homocysteic acid, linoleic acid, H_2_O_2_, polychlorinated biphenyls, pilocarpine, glutamate [2]–[7], and some diseases such as seizure and neurodegenerative diseases [5], [8]–[9]. Moreover, it has been shown that vitamin E can protect against cognitive and memory deficits induced by toxicants such as ozone, homocysteine, and ovariectomy and some diseases [10]–[14]. Alpha-tocopherol (α-tocopherol) can act as a chain-breaking antioxidant and a free radical scavenger, and protects cell membrane against oxidative damage by reacting with fatty acid peroxides via electron transfer [15]–[16]. Nevertheless, it has also been proven that treatment with relatively high concentrations of vitamin was neurotoxic [4], [17]. However, the underlying mechanisms for neurotoxicity from high concentrations of vitamin E are still unclear.

The free-living nematode *Caenorhabditis elegans* is one of the most thoroughly studied model animals, whose genome and its cell lineage have been well described [18]. Its experimental potential offers a system best suited for asking *in vivo* questions with relevance at the organism level, and many basic physiological processes, stress responses, signal transduction pathways, and epigenetic marks are conserved between *C. elegans* and humans [19]–[20]. So far, it has been proven that *C. elegans* is useful for toxicity assessment and toxicological studies from whole-animal level down to single cell level by serving as an alternative toxicity assay system for mammals [21]–[37]. Especially, *C. elegans* can be used for neurotoxicity evaluation and study of neurotoxicology with different relevant endpoints [38]–[68]. *C. elegans* has been already used to investigate the beneficial and adverse effects of vitamin E on animals. Administration of vitamin E could increase lifespan [1], [69]–[72], protect against oxidative stress during gametogenesis [73], and ameliorate cypermethrin-induced toxicity and oxidative stress in nematodes [74]. Moreover, treatment with vitamin E could retrieve and protect against UV-irradiation and metal exposure induced memory deficits in *C. elegans* [75]. In contrast to the beneficial effects of vitamin E on nematodes, treatment with 400 µg/mL of vitamin E had adverse effects on reproduction of nematodes, and possibly leads to growth retardation or developmental delay [69]. Administration of 400 µg/mL of vitamin E also shortens the extinction period of an associative learning so as to decrease memory behavior of nematodes [75]. However, the possible adverse effects of high concentration of vitamin E on perception and learning behavior and the underlying mechanisms are still unknown in *C. elegans*.

The nematode *C. elegans* is an attractive model organism to study learning and memory for its simple nervous system and ability to respond to diverse environmental stimuli [76]–[77]. In *C. elegans*, previous studies demonstrated that exposure to heavy metals can suppress learning behavior of animals [78]–[81]. To date, thermotaxis is one of the major paradigms used in associative learning research [76]. In this learning assay system, nematodes can be trained to move toward a temperature and trace it, and the final behavior changes will be the results of an interaction between temperature and food state [76]. Recording of this type of associative learning will be influenced by abnormal isothermal tracking (IT) and locomotion behaviors in the examined nematodes [82]. In the present study, we first explored *in vivo* assay system of *C. elegans* to investigate the possible adverse effects of high concentration of vitamin E on thermosensation and thermotaxis learning. Moreover, we further examined the underlying mechanisms explaining the toxicity formation for thermosensation and thermotaxis learning induced by high concentration of vitamin E in *C. elegans*.

## Results

### Effects of Vitamin E Treatment on Thermotaxis Learning in *C. elegans*

**Figure 1:**
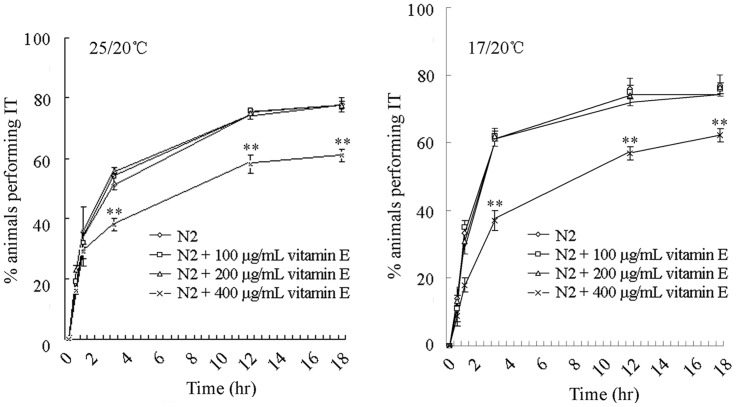
Effects of vitamin E treatment at different concentrations on thermotaxis learning behavior as monitored by 25/20°C or 17/20°C thermotaxis assays in *C. elegans*. IT, isothermotracking. Data are expressed as mean ± SEM. **p<0.01 vs. N2.

In the learning assay model, vitamin E treated nematodes were first cultured at 25 or 17°C, and then shifted to 20°C temperature condition for different time intervals. Based on the further evaluation of the ability of vitamin E treated nematodes to track a temperature of 20°C in a radial gradient, treatment with 100–200 µg/mL of vitamin E did not noticeably influence thermotaxis associative learning behavior at the assayed different time intervals compared with the control (Fig. 1). In contrast, treatment with 400 µg/mL of vitamin E significantly (*p*<0.01) decreased thermotaxis associative learning at the time intervals of 3, 12, and 18 hr compared with the control, although thermotaxis learning behaviors at the time intervals of 0.5 hr and 1 hr in nematodes exposed to 400 µg/mL vitamin E were similar to those of the control (Fig. 1). Therefore, treatment with vitamin E at the concentration of 400 µg/mL may reduce thermotaxis associative learning to a certain degree in *C. elegans*.

### Effects of Vitamin E Treatment on Thermosensation and Locomotion Behavior in *C. elegans*

**Figure 2:**
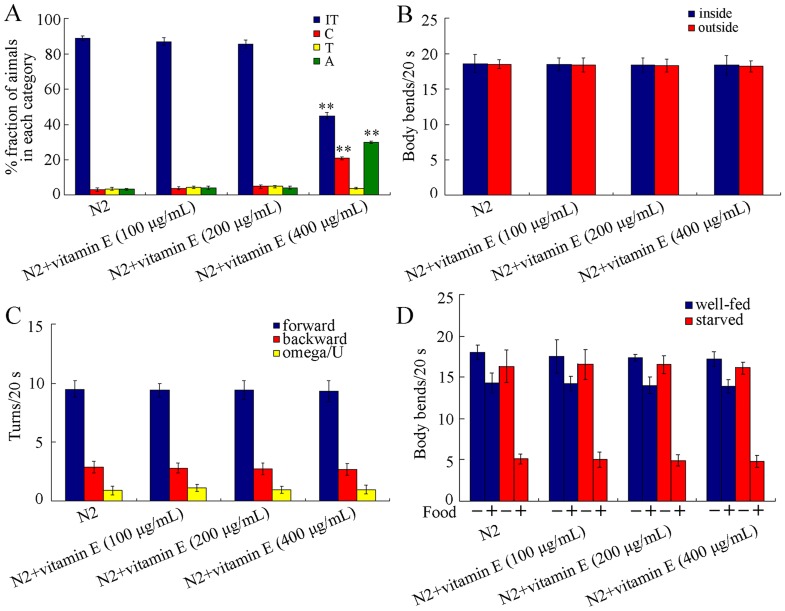
Effects of vitamin E treatment at different concentrations on thermotaxis and locomotion behaviors in *C. elegans*. (A) Effects of vitamin E treatment at different concentrations on thermotaxis behavior. In the thermotaxis assay system, movement to 25°C was scored as thermophilic (T); movement to 17°C was scored as cryophilic (C); movement across the thermal gradient (17°C/25°C) was scored as athermotactic (A); movement at 20°C was scored as isothermal tracking behavior (IT). (B) Effects of vitamin E treatment at different concentrations on body bends of nematodes inside and outside learning assay model. (C) Effects of vitamin E treatment at different concentrations on basic movements of the examined nematodes. (D) Effects of vitamin E treatment at different concentrations on basal and enhanced slowing responses of the examined nematodes. Data are expressed as mean ± SEM. **p<0.01 vs. N2.

Because recording of the learning behavior in the used assay system may be influenced by thermosensation and locomotion behavior of the examined nematodes, we next investigated the effects of vitamin E treatment at different concentrations on thermosensation in nematodes. In the thermotaxis assay system, movement to 25°C was scored as thermophilic (T); movement to 17°C was scored as cryophilic (C); movement across the thermal gradient (17°C/25°C) was scored as athermotactic (A); movement at 20°C was scored as isothermal tracking behavior (IT) [83]–[84]. Treatment with 100 and 200 µg/mL of vitamin E did not obviously affect the thermotaxis; however, treatment with 400 µg/mL vitamin E significantly (*p*<0.01) inhibited thermotaxis to cultivation temperature and induced abnormal thermotactic and cryophilic behaviors compared with control (Fig. 2A), implying that the observed decrease in thermotaxis learning behavior in 400 µg/mL of vitamin E treated nematodes may be partially due to the abnormal or decreased thermotactic perception.

Again, we investigated the effects of vitamin E treatment at different concentrations on locomotion behavior of the examined nematodes. Treatments with all the examined concentrations of vitamin E did not noticeably influence body bends of nematodes inside and outside the assay system for thermotaxis learning recording (Fig. 2B). Moreover, treatments with all the examined concentrations of vitamin E did not significantly alter the basic movements including forward turn, backward turns, and Omega/U turns of nematodes (Fig. 2C). Nematodes treated with all the examined concentrations of vitamin E also showed normal basic movements on food (data not shown). These data suggest that the observed deficits in thermosensation and thermotaxis learning behavior in 400 µg/mL of vitamin E treated nematodes may be not due to the alterations of locomotion behaviors of nematodes.

Well-fed nematodes will move slower in the presence of food than in the absence of food (basal slowing response, dopamine pathway), whereas starved nematodes will move much more slowly in the presence of food (enhanced slowing response, serotonin pathway) [85]. We further observed that nematodes treated with all the examined concentrations of vitamin E were normal in both basal and enhanced slowing responses (Fig. 2D), demonstrating that the nematodes treated with the examined concentrations of vitamin E exhibited the normal locomotion response to food and to starvation. That is, the observed decrease in thermotaxis learning behavior in 400 µg/mL of vitamin E treated nematodes may be also not due to the altered perception of food or starvation in nematodes.

### Effects of Vitamin E Treatment on Neuronal Development *in C. elegans*

**Figure 3:**
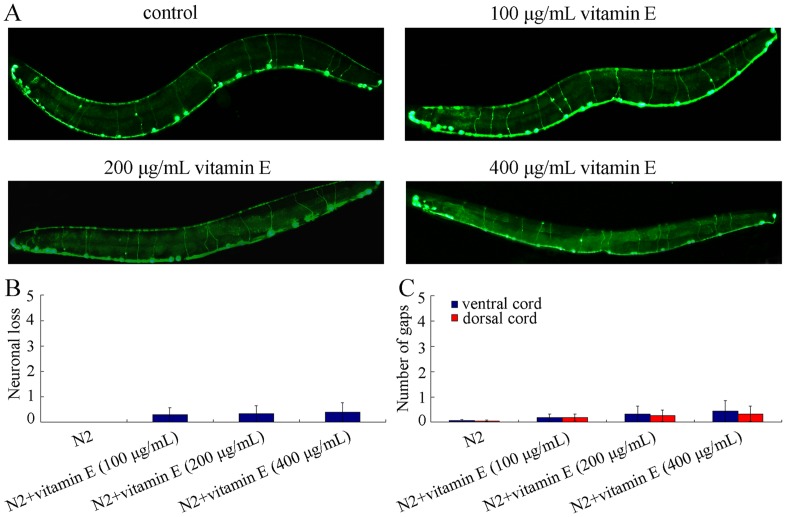
Effects of vitamin E treatment at different concentrations on development of GABAergic motor neurons in *C. elegans*. (A) Effects of vitamin E treatment on morphology of GABAergic motor neurons. The heads of nematodes were at the left of the images. (B) Effects of vitamin E treatment on neuronal loss of GABAergic motor neurons. (C) Effects of vitamin E treatment on dorsal/ventral cord gaps of GABAergic motor neurons. Data are expressed as mean ± SEM.

*oxIs12* is a fluorescent marker to label entire GABAergic motor neurons [52]. With the aid of the strain of *oxIs12*, we investigated the effects of vitamin E treatments on the development of the nervous system of nematodes. We observed that treatment with all the examined concentrations of vitamin E did not noticeably affect development of GABAergic motor neurons (Fig. 3A). That is, no noticeable axonal discontinuities and abnormal neuronal morphology of GABAergic motor neurons were found in vitamin E treated nematodes at the examined concentrations (Fig. 3A). Moreover, treatment with all the examined concentrations of vitamin E did not induce obvious neurodegeneration of nematodes, because no significant neuronal loss, and dorsal/ventral cord gaps were found in nematodes treated with all the examined concentrations of vitamin E (Figs. 3B and 3C).

**Figure 4:**
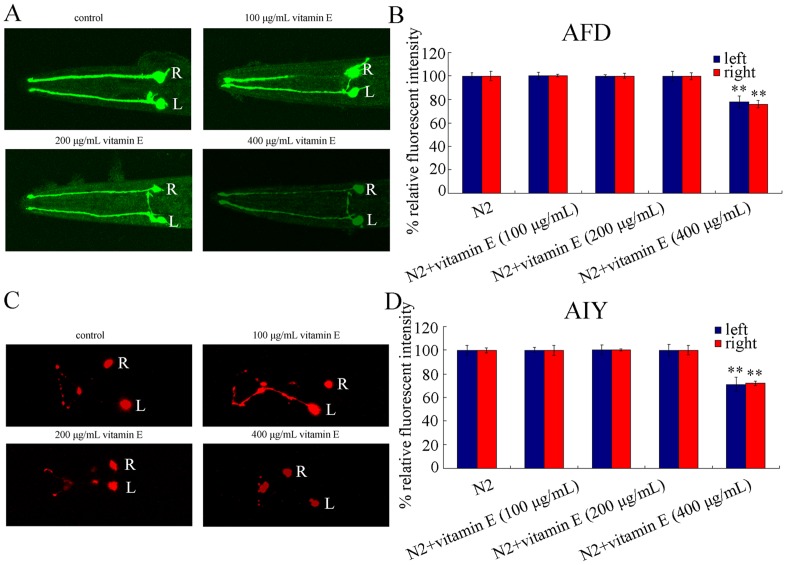
Effects of vitamin E treatment at different concentrations on development of AFD sensory neurons and AIY interneurons in *C. elegans*. (A) Effects of vitamin E treatment on morphology of AFD sensory neurons. (B) Effects of vitamin E treatment on fluorescent intensities of cell bodies in AFD sensory neurons. (C) Effects of vitamin E treatment on morphology of AIY interneurons. (D) Effects of vitamin E treatment on fluorescent intensities of cell bodies in AIY interneurons. L, left, R, right. Data are expressed as mean ± SEM. **p<0.01 vs. N2.

In *C. elegans*, AIY interneurons play a key role in regulating thermotaxis learning, and AFD sensory neurons play a key role for thermotactic perception [76], [86]. *adEx1267* and *otIs133* are transgenic fluorescent markers to label the AFD sensory neurons and AIY interneurons, respectively [47], [63], [87]. With the aid of *adEx1267* and *otIs133*strains, we investigated the effects of vitamin E treatment at different concentrations on the development of AFD and AIY neurons in nematodes. We observed that treatment with all the examined concentrations of vitamin E did not obviously alter the morphology of both AFD sensory neurons and AIY interneurons (Fig. 4). Similarly, treatment with 100 µg/mL and 200 µg/mL of vitamin E did not noticeably affect relative fluorescent intensities of cell bodies in both AFD sensory neurons and AIY interneurons (Fig. 4). Nevertheless, we observed that treatment with 400 µg/mL of vitamin E significantly (*p*<0.05) suppressed relative fluorescent intensities of cell bodies in both AFD sensory neurons and AIY interneurons (Fig. 4). Therefore, developmental alterations of AFD sensory neurons and AIY interneurons may be associated with the formation of deficits in thermosensation and thermotaxis learning induced by high concentration of vitamin E in nematodes.

### Effects of Vitamin E Treatment on Synaptic Transmission *in C. elegans*

**Figure 5:**
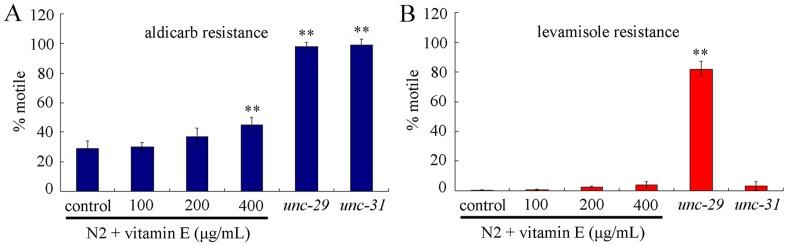
Effects of vitamin E treatment at different concentrations on synaptic neurotransmission in *C. elegans*. (A) Effects of vitamin E treatment on presynaptic function as evaluated by aldicarb resistance. (B) Effects of vitamin E treatment on postsynaptic function as evaluated by levamisole resistance. Data are expressed as mean ± SEM. **p<0.01 vs. control.

We further investigated the possibly altered synaptic functions in vitamin E treated nematodes at different concentrations. Drugs of aldicarb, an acetylcholinesterase (AChE) inhibitor, and levamisole, a nicotinic acetylcholine receptor (AChR) agonist, produce hyperactive cholinergic synapses, muscle hypercontraction, and paralysis [88]. Thus, synaptic transmission can be detected using aldicarb or levamisole, because nematodes lacking a functional AChR or defective in presynaptic Ca^2+^-dependent vesicle release are resistant to aldicarb, and nematodes only lacking a functional AChR are also resistant to levamisole [87]. Because strain *unc-29(e193)* is deficient for AChR, and strain *unc-31(e169)* is deficient for Ca^2+^-dependent activator protein for secretion (CAPS) [88], we used these two strains together with wild-type N2 as the controls. Based on assays of aldicarb and levamisole resistance, our data suggested that nematodes treated with 100 µg/mL and 200 µg/mL of vitamin E showed normal pre-synaptic and post-synaptic functions (Fig. 5A and 5B). Nematodes treated with 400 µg/mL of vitamin E also exhibited normal post-synaptic functions (Fig. 5B). However, nematodes treated with 400 µg/mL of vitamin E might have deficits in presynaptic function, because nematodes treated with 400 µg/mL of vitamin E exhibited moderately but significant (*p*<0.01) resistance to aldicarb compared with control (Fig. 5A). Therefore, besides development of AFD and AIY neurons, alterations of presynaptic neurotransmission may be also associated with the formation of deficits in thermosensation and thermotaxis learning in nematodes induced by high concentration of vitamin E.

### Promotion of Synaptic Transmission by Activating PKC-1 Effectively Retrieves the Altered Thermosensation and Thermotaxis Learning Induced by High Concentration of Vitamin E *in C. elegans*

**Figure 6:**
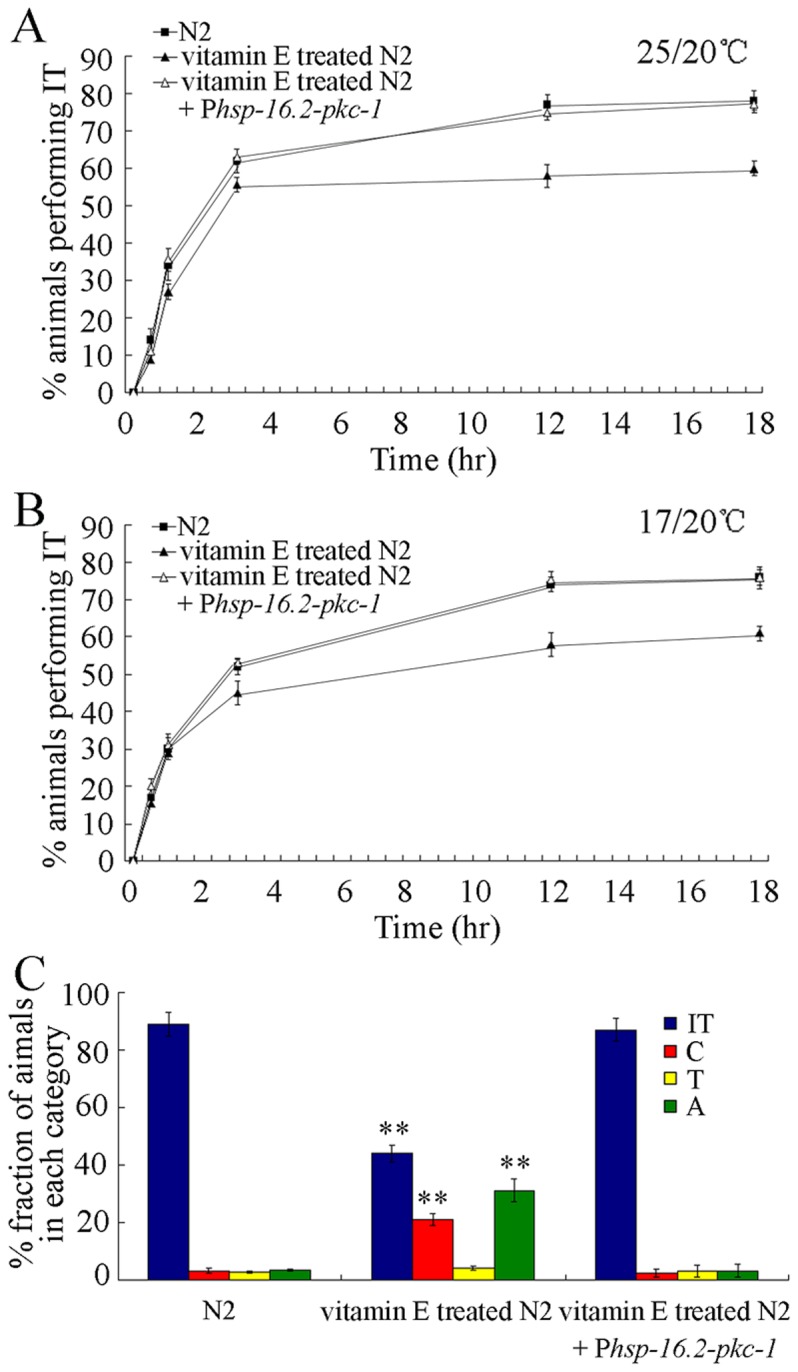
Promotion of synaptic transmission effectively retrieved deficits in learning and thermotaxis behaviors in nematodes treated with 400 µg/mL of vitamin E. (A) Promotion of synaptic transmission effectively retrieved deficits in thermotaxis learning behavior in nematodes treated with 400 µg/mL of vitamin E as monitored by 25/20°C thermotaxis assay. (B) Promotion of synaptic transmission effectively retrieved deficits in thermotaxis learning behavior in nematodes treated with 400 µg/mL of vitamin E as monitored by 17/20°C thermotaxis assay. (C) Promotion of synaptic transmission effectively retrieved deficits in thermotaxis in nematodes treated with 400 µg/mL of vitamin E. In the thermotaxis assay system, movement to 25°C was scored as thermophilic (T); movement to 17°C was scored as cryophilic (C); movement across the thermal gradient (17°C/25°C) was scored as athermotactic (A); movement at 20°C was scored as isothermal tracking behavior (IT). Data are expressed as mean ± SEM. **p<0.01 vs. N2.

In *C. elegans*, synaptic transmission can be promoted by expressing an active protein kinase C homologue (*pkc-1*(*gf*)) [89]–[90]. To induce expression of PKC-1, PKC-1 was activated using a heat shock promoter (P*hsp-16.2*) in nematodes [54]. After 400 µg/mL of vitamin E treatment, PKC-1 was activated by heat shock at 30°C for 4-hr. Wild-type N2 nematodes treated with heat-shock at 30°C for 4-hr showed normal locomotion behaviors [54]. Based on the evaluation of the ability to trace the temperature of 20°C for nematodes pre-conditioned at 25 or 17°C, we found that activation of PKC-1 effectively retrieved deficits in thermotaxis learning caused by treatment with 400 µg/mL of vitamin E (Figs. 6A and 6B). Moreover, activation of PKC-1 effectively retrieved deficits in thermotactic perception induced by treatment with 400 µg/mL of vitamin E (Fig. 6C). Therefore, promotion of synaptic transmission can effectively retrieve deficits in both thermosensation and thermotaxis learning in high concentration of vitamin E treated nematodes.

## Discussion

So far, most studies of vitamin E have focused on α-tocopherol supplementation, based on the rationale that this is the most abundant isoform in body [72]. α-tocopherol is a powerful liposoluble antioxidant and has many nonenzymatic actions [5]. In the current study, we focused on the adverse effects of α-tocopherol treatment on thermosensation and thermotaxis learning of nematodes and the underlying mechanisms. Previous study has demonstrated that the beneficial effects are usually from relatively low doses of vitamin E, and relatively high doses of vitamin E are often non-effective [91]–[92] or even neurotoxic [4], [17]. In *C. elegans*, besides thermotaxis memory behavior and reproduction [69], [75], both thermosensation and thermotaxis learning were also adversely affected by 400 µg/mL of vitamin E (Figs. 1 and 2). Similarly, treatment with vitamin E at the concentration of 400 µg/mL for 24-hr significantly decreased salt chemotaxis learning, but treatment with 100–200 µg/mL of vitamin E had no significant effects on salt chemotaxis learning of wild-type nematodes (data not shown). That is, under normal physiological conditions, relatively high concentration of vitamin administration will cause both reproductive and neuronal toxicity on *C. elegans*. Nevertheless, according to the data presented in this study, reduction or abnormality of thermotactic perception may be the primary defect in nematodes treated with high concentrations of vitamin E, whereas adverse effects on thermotaxis learning may be a secondary phenomenon in nematodes treated with high concentrations of vitamin E. Therefore, under normal physiological conditions, vitamin E should be carefully administrated.

Previous studies indicate that vitamin E may be involved in anti-cancer or cell death signaling [4]. In the current study, we observed that treatment with 400 µg/mL of vitamin E did not induce obvious neurodegeneration with the aid of GABAergic motor neuron marker (Fig. 3), suggesting that vitamin E at the concentration of 400 µg/mL was not involved in the activation of cell death signaling under normal physiological conditions.

In the present study, we raised two aspects of possible explanations for toxicity formation on thermosensation and thermotaxis learning induced by 400 µg/mL of vitamin E in *C. elegans*. The first possible explanation is the induction of deficits in neuronal development of AFD sensory neurons and AIY interneurons in nematodes exposed to 400 µg/mL of vitamin E (Fig. 4). Treatment with 400 µg/mL of vitamin E did not noticeably alter morphology of GABAergic motor neurons (Fig. 3). Moreover, treatment with 400 µg/mL of vitamin E also did not obviously influence the morphology of AFD sensory neurons and AIY interneurons (Fig. 4). Treatment with 400 µg/mL of vitamin E only moderately but significantly decreased fluorescent intensities of cell bodies in AFD sensory neurons and AIY interneurons (Fig. 4). Ablation of AFD sensory neurons caused nematodes to show cryophilic and abnormal thermotaxis phenotypes, and AIY-killed nematodes exhibited clear cryophilic phenotype [93]. Our data further demonstrated that treatment with 400 µg/mL vitamin E caused approximately 21% of the examined nematodes to show cryophilic phenotype and approximately 30% of the examined nematodes to exhibit abnormal thermotaxis phenotype (Fig. 2A). In addition, our data also imply that AFD sensory neurons and AIY interneurons may be somewhat more sensitive than motor neurons for assessing the possible adverse effects of vitamin E.

The second possible explanation is the formation of deficits in presynaptic transmission in nematodes exposed to 400 µg/mL of vitamin E (Fig. 5). Only moderate but significant resistance to aldicarb was observed in nematodes exposed to 400 µg/mL of vitamin E (Fig. 5A). One possibility is that toxicity of vitamin E at the concentration of 400 µg/mL on neurons of nematodes may be still very limited. Another possibility is that not structures and functions of all neurons may be affected by vitamin E at the concentration of 400 µg/mL. Furthermore, we found that vitamine E treatment at the concentration of 400 µg/mL only influenced presynaptic functions, but had no significant effects on postsynaptic functions of nematodes (Fig. 5), which is largely different from adverse effects of heavy metals on synaptic function in *C. elegans* [50].

The important role of synaptic function in regulating toxicity formation on thermosensation and thermotaxis learning caused by vitamin E treatment at the concentration of 400 µg/mL was confirmed by activating PKC-1 protein after vitamin E treatment. After 400 µg/mL of vitamin E treatment, activation of PKC-1 effectively retrieved deficits in both thermosensation and thermotaxis learning in nematodes pre-exposed to 400 µg/mL of vitamin E (Fig. 6). In the present study, our results indicate that most of the animals exposed to 400 µg/mL of vitamin E showed athermotactic behavior, and such a deficit could be rescued by PKC-1 expression (Figs. 2 and 6). In *C. elegans*, PKC-1 is thought to cause inactivation of AFD sensory neurons and loss of PKC-1 function leads to thermophilic drive [94]. Thus, treatment with high concentrations of vitamin E may exert adverse effects on thermosensation behavior in nematodes via inhibiting activity of PKC-1 in thermotaxis neurons. These data further imply that presynaptic function, together with structural alterations of AFD sensory neurons and AIY interneurons, may serve as useful biomarkers for detecting the potential adverse effects of high concentration of vitamin E. To retrieve neurotoxicity from high concentration of vitamin E, the potential drugs are suggested to be able to rescue or retrieve damage on synaptic functions.

In conclusion, in the present study, our data demonstrated that high concentration of vitamin E treatment from L1-larvae to young adult resulted in deficits in thermosensation and thermotaxis learning under normal physiological conditions in *C. elegans*. Two aspects of possibilities, abnormal neuronal development and abnormal synaptic function, were raised to explain the toxicity formation on thermosensation and thermotaxis learning in nematodes treated with relatively high concentration of vitamin E. Based on our observations, on the one hand, our data imply that vitamin E should be carefully administrated under normal physiological conditions. On the other hand, safety concentrations of vitamin E administration for other animals or human beings under normal physiological conditions still need to be carefully investigated, because *C. elegans* may be somewhat more sensitive than other assay systems for toxicity assessment.

## Materials and Methods

### Reagents

Vitamin E (α-tocopherol) was dissolved in ethanol, and then diluted into three concentrations (100, 200, and 400 µg/mL) as previously described [75]. All the other chemicals were obtained from Sigma-Aldrich (St. Louis, MO, USA).

### Strain Preparation

Nematodes used in the present study were wild-type N2, *unc-29(e193)*, *unc-31(e169)*, *oxIs12*[*Is*(P*unc-47::GFP*)], *adEx1267*[*Ex*(P*gcy-8::GFP*)], *otIs133*[*Is*(P*ttx-3::RFP*)], originally obtained from *Caenorhabditis* Genetics Center (funded by the NIH National Center for Research Resource, USA), and *Ex*(P*hsp-16.2-pkc-1*). They were maintained on nematode growth medium (NGM) plates seeded with *Escherichia* OP50 at 20°C as described [95]. Gravid animals were washed off the plates into centrifuge tubes and were lysed with a bleaching mixture (0.45 M NaOH, 2% HOCl). Age synchronous populations of larva (L1-stage) were obtained by collection as described [22]. L1-stage larval animals were washed with double-distilled water, followed by washing with K medium (50 mM NaCl, 30 mM KCl, 10 mM NaOAc, pH 5.5) [96]. Exposures were performed in 12-well sterile tissue culture plates. All exposures were performed from L1-larvae to young adult in 20°C incubator in the presence of food.

### Locomotion Behavior Assay

To assay body bend frequency, nematodes were picked onto a NGM plate and scored for number of body bends in an interval of 20 sec. A body bend was counted as a change in the direction of the part of the animals corresponding to the posterior bulb of the pharynx along *y* axis, assuming that animal was traveling along *x* axis. In the learning assay model, nematodes within the 20°C region at the 18^th^-hr time point were picked out for the body bend assay. Thirty nematodes were examined per treatment.

Three basic movements, forward sinusoidal movement (forward turns), reversal movement (backward turns), and turns in which nematodes change direction (Omega/U turns) of bodies in a 20-sec interval were measured on or off food. The method was basically performed as described previously [97]–[98]. The examined nematodes were picked onto a NGM plate with or without food and scored for number of forward turn, backward turn, or Omega turns in an interval of 20 sec. In Omega turns, a nematode’ head touches the tail, whereas angle of the body is typically >90° in U turns. Thirty nematodes were examined per treatment.

Basal slowing response and enhanced slowing response of nematodes were examined as described [85].

### Thermotaxis Assay

Procedure for the themotaxis assay using a radial temperature gradient was performed according to previous descriptions [93], [99]. A radial thermal gradient will be created on an agar surface in the 9-cm Petri dish, in which a steeper gradient, ranging from approximate 17°C at the central area to approximate 25°C at the periphery, is formed. A radial gradient of temperature was created by placing a vial containing frozen acetic acid on the bottom of the plate and incubating the plate at 26°C for 90-min in the presence of a constant humidity of 60%. The examined nematodes were raised in the presence of food at 20°C. Nematodes were then transferred onto a fresh plate devoid of bacteria for 2-min. Individual nematodes were deposited on a 9-cm Petri dish with a thermal gradient, and allowed to move freely for 1.5–2 h. Upon removal of the nematode from the plats, tracks left on the agar surface were photographed. Each data point represents 3 independent assays using 30 nematodes per treatment.

### Thermotaxis Tracking Behavior Assay for Learning

Learning assay was performed basically as previously described [81]–[82]. Approximately 50 examined nematodes were grown at 25 or 17°C for 12-hr in the presence of food on a 9-cm Petri dish, and then shifted individually to a selected plate at 20°C for different time intervals (0, 0.5, 1, 3, 12, and 18 hr). The aim of this assay model was to investigate the abilities of nematodes to learn new cultivation temperature (20°C), which was different from their original cultivation temperature (25 or 17°C). Then the examined nematodes were analyzed for their IT behaviors as described above. Three replicates were performed.

### Pharmacological Assay

Aldicarb and levamisole resistance were examined basically as described [87]. Approximately 50 examined nematodes were placed on freshly seeded NGM plates containing 1 mM aldicarb or 100 µM levamisole. After 8 hr and 2 hr, respectively, nematodes were scored as motile if they still exhibited locomotion and pharyngeal pumping when continuously prodded three times. Locomotion movement was scored as positive if any obvious body bend could be detected and any body wall muscle activity could be observed, and pharyngeal pumping was scored as positive if nematodes demonstrated it continuously during a 1-min period. Three replicates were performed.

### Fluorescent Images of Neurons

Acquisition of series of images was performed in a Leica TCS-NT confocal laser scanning microscope (Leica Microsystems, Heidelberg, Germany) equipped with an Argon/Krypton gas laser. In the instrument used in this study, a Cy2/Alexa Fluor 488 or ‘green’ channel and an Alexa Fluor 594 or ‘red’ channel had been configured. Relative intensities of fluorescent puncta for cell bodies of AFD and AIY neurons were examined in at least 20 nematodes.

### DNA Construct and Germline Transformation

Full length of *pkc-1 *cDNA was subcloned into the site of BamHI/KpnI of pPD49_78 vector behind promoter fragment of *hsp-16.2* gene. Vector of pPD49_78 contains a promoter fragment of *hsp-16.2* gene, which was used for induce expression of PKC-1 by treating nematodes at 30°C for 4-hr [54]. Germline transformation was performed as described [100] by coinjecting testing DNA at a concentration of 20 µg/mL and a transgenic marker of P*dop-1::GFP* into gonad of nematodes. Transgenic nematodes were heat shocked at 30°C for 4-hr after 400 µg/mL of vitamin E treatment, and the examined transgenic nematodes were cultured at 20°C again.

### Statistical Analysis

All data in this article were expressed as means ± standard error of the mean (S.E.M.). Graphs were generated using Microsoft Excel (Microsoft Corp., Redmond, WA). Statistical analysis was performed using SPSS 12.0 (SPSS Inc., Chicago, USA). Differences between groups were determined using analysis of variance (ANOVA). Probability levels of 0.05 and 0.01 were considered statistically significant.
